# Health status, healthcare utilisation, and quality of life among the coastal communities in Sabah: Protocol of a population-based survey

**DOI:** 10.1097/MD.0000000000022067

**Published:** 2020-09-11

**Authors:** Maznah Dahlui, Amirah Azzeri, Mohd Aizat Zain, Mohd Iqbal Mohd Noor, Hafiz Jaafar, Amy Yee Hui Then, Julia Suhaimi, Fatimah Kari, Lota A. Creencia, John Roderick Madarcos, Edgar Jose, Lora E. Fleming, Mathew P. White, Karyn Morrissey, Kamal Solhaimi Fadzil, Hong Ching Goh

**Affiliations:** a Centre for Population Health (CePH), Department of Social and Preventive Medicine, Faculty of Medicine, University of Malaya; b Faculty of Public Health, University of Airlangga, Surabaya, Indonesia; c Department of Primary Care, Faculty of Medicine and Health Sciences, Universiti Sains Islam Malaysia; d Department of Urban and Regional Planning, Faculty of Built Environment, University of Malaya; e Faculty of Business Management, Universiti Teknologi MARA, 26400, Pahang, Malaysia; f Institute of Biological Science, Faculty of Science, University of Malaya; g Department of Primary Care Medicine, Faculty of Medicine, University of Malaya; h Department of Economics, Faculty of Economics and Administration, University of Malaya, Kuala Lumpur, Malaysia; i College of Fisheries and Aquatic Sciences, Western Philippines University, Puerto Princesa City, Palawan, Philippines; j European Centre for Environment and Human Health, University of Exeter Medical School, Knowledge Spa, Royal Cornwall Hospital, Truro, Cornwall, TR1 3LJ, UK; k Department of Anthropology, Faculty of Arts and Social Sciences, University of Malaya, Kuala Lumpur, Malaysia.

**Keywords:** cross-sectional, population, questionnaire and survey, seaside, well-being

## Abstract

**Introduction:**

Coastal areas in Malaysia can have important impacts on the livelihoods and health of local communities. Efforts by Malaysian government to develop and improve the landscape and ecosystem have been planned; however, the progress has been relatively slow because some of the coastal areas are remote and relatively inaccessible. Thus, these coastal communities face various challenges in health, healthcare and quality of life. This paper presents a study protocol to examine the health status, healthcare utilisation, and quality of life among the coastal communities. In addition, the relationship between the community and their coastal environment is examined.

**Methodology and analysis:**

The population of interest is the coastal communities residing within the Tun Mustapha Park in Sabah, Malaysia. The data collection is planned for a duration of 6 months and the findings are expected by December 2020. A random cluster sampling will be conducted at three districts of Sabah. This study will collect 600 adult respondents (300 households are estimated to be collected) at age of 18 and above. The project is a cross sectional study via face-to-face interview with administered questionnaires, anthropometrics measurements and observation of the living condition performed by trained interviewers.

## Abstract 2

Supplemental Digital Content is available in the text

## 1 Introduction

The coastal environment has an important relationship with the human health and quality of life among the coastal communities. The environment influences the health of the population in various ways both positively and negatively. Several studies revealed that living close to the coastline encourages people to engage in recreational activities, which improves physical and psychological quality of life.^[1–5]^ Living in the coastal environment is also associated with higher quality of life and better mental health status due to improved socialization and more leisure activities.^[1–3,5]^

Nevertheless, there are several factors contributing to the potential negative impacts on the people living in the coastal environment. Ecological factors such as extreme natural calamities, habitat modification, and water pollution in these areas can lead to serious diseases, injuries and inadequate nutrition.^[6–8]^ In addition, due to the remoteness, many coastal areas globally are underdeveloped and have not received enough attention from the local or national government even though many potential industrial resources are available. This has led to a slow rate of industrial activities and infrastructure development.^[9]^

One of the least developed infrastructures in many remote coastal areas is the provision of healthcare. The lack of progress in healthcare facilities and other associated resources has resulted in difficult access to these services, low health awareness and health inequalities between coastal, non-coastal and urban population.^[10,11]^ These, in turn, can result in poor health and poor quality of life among the coastal communities, which may lead to numerous negative health consequences.

Studies have shown that several factors such as densely populated areas, poor housing condition with lack of supply to clean water, and improper waste disposal systems have exposed these populations to many infectious diseases including tuberculosis and parasitic infections.^[12–14]^ Furthermore, inadequate access to healthcare services and limited health knowledge are associated with a higher burden of chronic diseases (e.g. cardiovascular and hypertension^[15]^), and a high prevalence of tobacco, alcohol and illegal drug uses^[12,16,17]^ among coastal communities.

Due to the geographical location of Malaysia, coastal areas have an important influence on the livelihoods and health of the population. The coastal ecosystem in Malaysia supports a large proportion of the population by provision of significant resources for important industries such as urbanization, agriculture, fisheries, aquaculture, oil and gas exploitation, transportation, communication and recreation, all of which are linked to health benefits and risks of the community.^[18,19]^

In many local coastal areas, efforts to develop and improve the landscape and ecosystem have been planned extensively especially in Peninsular Malaysia.^[18–21]^ In contrast, the socioeconomic progress in Sabah has been relatively slow, mainly because the coastal zones in Sabah are isolated and inaccessible, as well as having language diversity and cultural differences.^[22]^ Even though public health facilities are available, they are limited and might not reach those in need due to insufficient infrastructure to access the services.^[10]^ In addition, Sabah is facing a high rate of poverty^[10]^ and an large influx of stateless and undocumented people in its coastal areas.^[23]^ Because of these factors, the coastal communities in Sabah are facing various health and healthcare challenges.

To date, research focusing on the health issues, healthcare gaps and health needs of people at coastal areas of Sabah are lacking. Therefore, this paper presents a study protocol to examine the health status, healthcare utilisation and quality of life of coastal communities in Sabah. In addition, the study protocol potentially could also be replicated in other remote coastal communities in the South-East Asian region.

## 2 Methodology

### 2.1 Study background

This study is one of the 12 projects under “Blue Communities” grant (https://www.blue-communities.org). The Blue Communities is a 4-year research capacity-building program focused on marine planning, food security and human health and well-being in marine protected areas located in South-East Asia, funded by Global Challenges Research Fund of United Kingdom Research and Innovation. The present study protocol is specifically developed for selected coastal communities in Malaysia.

This is a cross-sectional study to determine health status, healthcare utilisation, and quality of life among the coastal communities in Sabah. It will be conducted for a duration of 6 months. Subsequent intervention studies will be conducted based on the findings of this study. The population of interest is the coastal communities that reside within the Tun Mustapha Park (TMP) boundary in Sabah. TMP was chosen because it is the largest marine protected area recently gazette in Malaysia; and the first multiple-use zoning areas, which was placed under joint-management program involving collaboration between the Sabah Park authorities, World Wildlife Fund (WWF) Malaysia, and local communities.^[24]^ The joint-management program aimed to protect marine and coastal ecosystems as well as to manage the rich resources contained within it, which will subsequently benefit and support the quality of life among the 80,000 people living in the area.

Presently, TMP had been experiencing several environmental issues that can affect the human communities. It is under threat from habitat degradation and overfishing which will decrease the available marine resources resulting in negative impacts on the health and quality of life of the community.^[24]^ Given the inadequate health-linked research, community health needs and potential risks of living in the coastal communities, this study is proposed to address these gaps by taking the unique perspective of examining linkages to the coastal and marine environment of these communities.

### 2.2 Study site and sampling procedure

**Figure 1:**
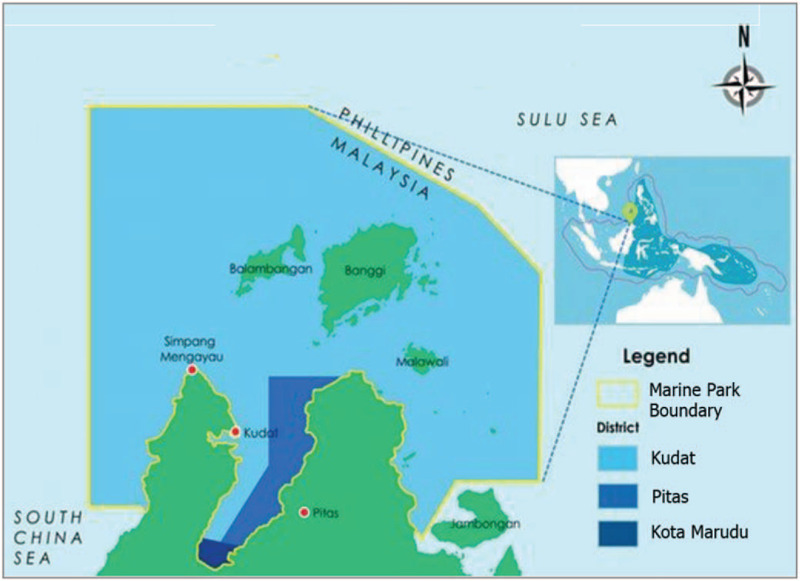
The marine park boundary of the Kudat, Pitas and Kota Marudu district regions in the Tun Mustapha Park.

In this study, only villages situated within 5 km radius from coastline are included based on the standard definition of coastal area.^[19]^ All villages included in this study are registered under the Sabah Parks supervision.^[24]^ The villages are all from three districts, within TMP, namely Kudat, Kota Marudu, and Pitas (Fig. 1). All the villages will be identified and further divided into several units based on its density. One unit consists of approximately 50 houses. This is to avoid the overlapping of household coverage due to the high density of the villages. A random cluster sampling (based on unit) will be conducted at all the three districts (Kudat, Kota Marudu, and Pitas). All houses in the selected units will be visited. Following that, all adult households (adult is defined as individual at the age of 18 years old or above) will be recruited as participants upon their consent (see Supplemental Digital Content (Appendix 1), which describes on the informed consent form).

### 2.3 Sampling size

Sample size calculation is performed using an Open Epi Software version 3.01. Based on the latest National Health and Morbidity Survey (NHMS) 2015, the overall disease prevalence of hypertension among 18 years and above in Malaysia is 30.3%. Using the overall hypertension in Malaysia of 30.3%,^[25]^ the minimum sample size calculated for this study is 561 people for 99% confidence level. Adding approximately 10% of drop out probability, the total sample size needed for this study is about 600 people.

Therefore, this study will involve a collection of 600 adults. We estimate that from 600 adults, there will be about 300 households to be interviewed based on the assumption that 1 household has of 2 adults. To ensure that the districts in TMP are well represented, the percentage of population selected is determined to be at 50%, 30% and 20% for Kudat, Kota Marudu and Pitas from the total number of populations of these three districts, respectively. Therefore, from the 600 adults, 300 adults will be sampled from Kudat (from three randomly selected units), 180 adults (from 2 randomly selected units) from Kota Marudu and the remaining 120 adults (from 1 randomly selected unit) will be from Pitas.

### 2.4 Tools and instruments

The main tools and instruments of data collection are questionnaires which also include a form for physical examination and observation. In this study, the questionnaire entitled “Blue Communities Project 6 Household Survey” (see Supplemental Digital Content (Questionnaire), that will be used for data collection) will be used. A pilot study to validate this questionnaire was conducted among the selected population in TMP prior to actual data collection. There are 2 versions of the questionnaire: one with Part A to Part I for the head of household; and another one with only Part C to Part I for other adult household members who are willing to join the survey. All participants must be 18 years old and above during time of the visit. The 9 parts of the full questionnaire are as follows:

- Part A: Housing and the environment (Head of household only)
  Questions in Part A will capture the characteristic of the household, housing infrastructure, surrounding area, excreta disposal, basic needs and basic amenities in the house.
- Part B: Household income (Head of household only)
  Questions in Part B will capture data on annual household income of the household from all possible income sources.
- Part C: Socio-demographic (All household members including head of household)
  Questions in Part C will capture sociodemographic information such as date of birth, ethnicity, academic background, employment status, relationship status, and other relevant information.
- Part D: Expenditure and utilization of healthcare goods and services (All household members including head of household)
  Questions in Part D will capture data on source of health payments, healthcare service utilisation, traditional and complementary medicine practice, and healthcare expenditure incurred.
- Part E: Modifiable lifestyle factors (All household members including head of household)
  Questions in Part E will capture information on tobacco consumption, nutritional intake, physical activity, medical condition and injury history.
- Part F: SF-12 quality of life (All household members including head of household)
  Questions in Part F will assess the quality of life of the study participant with the use of standard and generic quality of life assessment tool.
- Part G: Interaction with local marine environment
  Questions in Part G will capture the types of activities and exposures of the participants in the coastal area of TMP.
- Part H: Perceptions of the marine environment in TMP (All household members including head of household)
  Questions in Part H will elucidate the perceptions and awareness of the respondents on the drivers and pressures in marine environment in TMP.
- Part I: Medical proforma (All household members including head of household)
  Part I will assess the anthropometric measurement and blood pressure of the study participant. The anthropometric measurements that will be taken include height, weight, waist circumference and hip circumference.

Other study tools that will be used in this survey are calibrated OMRON M2 HEM-7120 Automatic BP Monitor to measure respondents blood pressure, calibrated vertical SECA Portable 217 Stadiometer to measure respondents height, calibrated SECA 813 digital electronic weighing scale to measure respondent weight and SECA 201 Ergonomic measuring tape to measure respondent waist (abdominal) and hip circumferences.

For blood pressure measurement, it will be categorised as normotensive, hypotensive, or hypertensive based on standard definitions used in the recent clinical practice guideline for management of hypertension in Malaysia.^[26]^ The reading will be taken while sitting. Two readings will be taken for each study participant. Second reading will be taken, 1 minute after the first blood pressure measurement. If both are normal, the average of the readings will be documented. If either the first or the second readings is abnormal, the interviewer will proceed with third reading, 30 minutes after the second reading. If the third reading is high, then a referral letter to the nearest hospital will be given to the study participant. If the third reading of is normotensive, then the blood pressure finding will be recorded. However, the interviewers will advise the participants to double-check his/her blood pressure at the clinic/hospital in the future. All the readings will be recorded in the questionnaire answer booklet.

### 2.5 Data collection

Prior to data collection, researchers will have to conduct ground-truthing of the study survey sites. This process is crucial to ensure that information provided is the same as that obtained on the ground. Information on study sites such as map of the selected units, number of houses in the units, and accessibility of transport to the units can be obtained from the local district office. An official letter for permission to acquire this information will be submitted to the local district offices. Once the information has been collected, the researchers will be in contact with the head of villages (known locally as *Ketua Kampung* or sometimes referred to as *Ketua Adat*) for the selected units.

Researchers will conduct the ground-truthing together with the head of village to cross-check the information provided by the local district offices (if made available). If the village head is not available for ground-truthing, researchers must acquire permission from the village head to get help from local research liaison officers in Kudat, Kota Marudu and Pitas. During the ground-truth, the 3 most crucial observations that the project manager must acquire are as follows: the layout of the houses in the village (scattered randomly, next to each other or etc.), the population density in the village, and the transport accessibility to the village study site.

The data collection is planned for a duration of 6 months and the findings are expected by December 2020 to plan for a subsequent health intervention programme. The main method of data collection is of administered questionnaire. The observation of house condition, its surrounding area and an individual physical examination will be conducted. Since the survey will involve multiple locations at TMP, it is estimated that 4 to 6 interviewers will be required during the fieldwork. The interviewers will be teamed in a group of 2 people. It is highly advisable to have one (1) male and one (1) female for each pair and at least one of the pair from the local community for security and dialect reasons. Researchers will then assign each pair to conduct the household survey at the selected villages using the prepared schedule.

Ground staff (from among the local community in each data collection site) who acts as a liaison officer will be appointed. Prior to data collection, the ground staff will introduce the interviewers to the household members to ensure that the head and members of the household are present and give permission to enter their personal premise. Once the permission obtained, the interviewer will explain regarding the nature of study based on the participant's information sheet (see Supplemental Digital Content (Appendix 2), which describes the participant's information sheet); afterward, the information sheet will be given to the participant(s) for reference. In addition, the interviewer will reassure the participants that the information given in the study will remain confidential and their participation is entirely voluntary.

The face-to-face interview will begin shortly after the head and members of household have signed the consent form. All adults in the household that give their consent will be interviewed. Blood pressure and anthropometric measurements (including height, weight, waist and hip circumference), will be taken during the face-to-face interview depending on the suitability and participant's preference.

In the case where the head of the household is not present during the visit, the house will be marked in the map and a second visit will be arranged for the next day. However, the house will be excluded if the head of the household is still not present during the second visit.

### 2.6 Data management and analysis

#### 2.6.1 Safe keeping of the survey questionnaires

At the end of each day of data collection, the project manager will gather all interviewers and conduct post-mortem session. Any loopholes identified during the process must be addressed immediately to avoid further mistakes and invalid data collected. In addition, the project manager will verify if the number of answer booklets tallies with the number of households surveyed. Upon verification, the answer booklets will be placed in a secure box, while the consent form will be kept in a separate secure box to avoid any breach of information. Only the project manager and the Principal Investigator have access right to these secured boxes.

#### 2.6.2 Data entry and analysis

Data entry is to be completed within 2 to 4 weeks after the completion of groundwork depending on the number of respondents. Once the data have been completely entered in the IBM SPSS version 24, a random cross-checking on the dataset will be performed to maintain data accuracy. The cross-checking will be set at 20% of the overall questionnaires entered. Data analysis will be divided into descriptive and analytical analysis. Descriptive analysis will be performed on the socio-demographic characteristics, anthropometry measurements, expenditure and utilisation of healthcare goods and services, modifiable lifestyle factors and reported quality of life among the study population. Analytical analysis will be conducted to determine the association between the socio-demographic characteristics, health status and modifiable risk factors with the quality of life and healthcare utilisation. Analysis of results will be done using IBM SPSS version 24. The data and results will be presented to and validated by relevant stakeholders and Blue Communities research group members in the form of reports and seminar sharing session

#### 2.6.3 Ethics and dissemination

The present study has been given ethics approval by the University of Malaya Research Ethics Committee with a reference number of UM.TNC2/UMREC-522. Written informed consent will be obtained from all the respondents prior the interview. The results in the present study will be disseminated in peer-reviewed journal, academic conference, and stakeholder seminar.

## 3 Discussion

Previous studies have reported that the coastal communities in Sabah are facing several health and healthcare challenges. These included a nutritional inadequacy among children and adolescents in Sabah, either moderately or severely underweight, especially those who reside in the interior and coastal areas of Sabah.^[27]^ Lack of access to nutritional foods and poor knowledge on healthy diets were found to be the main risk factors for the problem.^[27]^ In addition, in a previous report, there were 30% to 60% of people in rural coastal areas in Sabah who were unaware that they had several non-communicable diseases and were at risk for developing cardiovascular diseases.^[10]^ This appeared to be mainly due to low awareness of routine health check-up and poor access to healthcare services particularly in the rural coastal areas.^[10]^

Malaria remains a significant public health problem in Sabah, with an incidence of 61.3 per 100,000 population and 23% among children less than 18 years of age.^[27]^ The majority of cases in Sabah are due to a type of plasmodium which is *Plasmodium knowlesi*.^[14,28,29]^ Recently, *Plasmodium knowlesi* has emerged as a common and potentially fatal cause of human malaria in Malaysian Borneo. The main factors for malarial infection at coastal areas in Sabah are found to be poor housing construction, occupational exposure and seasonal factors.^[14]^

On the other hand, Sabah also contributed to the highest number of tuberculosis cases in Malaysia. The influx of immigrants from neighbouring countries (Philippines and Indonesia) to Sabah appeared to have contributed to the annual cases in the states since 1982.^[30]^ The cases detected among the immigrants in Malaysia are highest in Sabah, which accounts for at least 24% of the total annual cases detected. People of the Kadazan, Dusun and Murut (KDM) tribal communities appeared to be highly susceptible to the infection; and most of the KDM live in the interior parts of Sabah such as Kota Marudu, Pitas, Kota Belud and Pensiangan.^[30]^

The health status, healthcare utilisation and quality of life of coastal communities at Tun Mustapha Park, Kudat Sabah are the focal points of this study. This study aims to identify the factors that hinder the coastal communities in Sabah from accessing and receiving appropriate healthcare services. The information gathered here will be used to design an effective intervention to improve health status and improve quality of life of the community.

## 4 Conclusion

In conclusion, this study will determine the health status, healthcare utilization and quality of life of the coastal communities in Sabah. This study has the potential to increase awareness of health inequalities among these communities and to develop an intervention which was tailor according to the context-specific health and quality of life intervention program in the community. The experience and information generated by this study in these aspects should be of interest at the national and international level to improve the overall health and quality of life among the coastal communities, not only in the Sabah, but also in the South East Asia region.

## Acknowledgments

We are extremely grateful to colleagues within the Blue Communities project for their support. These include colleagues at the University of Exeter and Western Philippines University, who supported this work in developing parts G and H of the questionnaire. This work has received funding in part from the Global Challenges Research Fund (GCRF) via the United Kingdom Research and Innovation (UKRI) under grant agreement reference NE/P021107/1 to the Blue Communities project and University of Malaya (IF052-2017).

## Author contributions

**Conceptualization**: Maznah Dahlui

**Data collection**: Mohd Aizat Zain, Mohd Iqbal Mohd Noor

**Methodology:** Mohd Aizat Zain, Mohd Iqbal Mohd Noor, Kamal Solhaimi Fadzil, Amirah Azzeri, Julia Suhaimi, Fatimah Kari, Hafiz Jaafar

**Funding acquisition:** Hong Ching Goh, Amy Yee Hui Then

**Questionnaire development:** Maznah Dahlui, Lota A Creencia, John Roderick Madarcos, Edgar Jose, Amirah Azzeri, Karyn Morrissey

**Supervision:** Maznah Dahlui, Hong Ching Goh, Amy Yee Hui Then

**Writing – original draft**: Amirah Azzeri

**Writing – review & editing:** Mohd Aizat Zain, Mohd Iqbal Mohd Noor, Maznah Dahlui, Amy Yee Hui Then, Hong Ching Goh, Lora E Fleming, Mathew P White

## Supplementary Material

Supplemental Digital Content

## Supplementary Material

Supplemental Digital Content

## Supplementary Material

Supplemental Digital Content
