# Supplementary material for: Health status, healthcare utilisation, and quality of life among the coastal communities in Sabah: Protocol of a population-based survey
Source: Medicine (Baltimore). 2020 Sep 11;99(37):e22067. doi: 10.1097/MD.0000000000022067 (PMC7489655; doi:10.1097/MD.0000000000022067)
Supplement: Supplemental Digital Content [file medi-99-e22067-s001.docx]

**BORANG PERSETUJUAN UNTUK SOAL SELIDIK**

**Tajuk Penyelidikan:** “Projek 6: Faedah Kesejahteraan dan Risiko Persisiran Kehidupan Pantai”

Dengan mengisi borang ini, saya mengesahkan bahawa penyelidik telah memberi taklimat mengenai penyelidikan ini dan maklumat peserta kepada saya, dan saya secara sukarela bersetuju untuk mengambil bahagian dalam penyelidikan ini dan akan memberi maklumat kepada penyiasat seperti yang diminta. Saya tahu bahawa saya mempunyai hak untuk menarik diri pada bila-bila masa sepanjang tempoh kajian dijalankan. Saya juga memberi perakuan bahawa saya telah menerima wang saguhati diatas penyertaan dalam kajian ini berjumlah RM 10.00 tunai.

**Kerahsiaan:**

- Jawapan dan maklumat anda akan dirahsiakan oleh penyelidik dan tidak akan dimaklumkan kepada orang awam melainkan dikehendaki oleh undang-undang.

Dengan menandatangani borang persetujuan ini, anda akan membenarkan rekod anda dikaji, dianalisis dan data yang timbul daripada kajian ini akan digunakan untuk tujuan penyelidikan.

Tandatangan Peserta: __________________________

Tarikh: _______________________________________

Nama Penuh: _________________________________

Nama Kampung & No.Rumah: ____________________

Tandatangan Saksi: ___________________________

Nama: ______________________________________

Nombor unit peserta: __________________________

Tandatangan Penyelidik: _______________________

Nama: ______________________________________
