# Supplementary material for: Health status, healthcare utilisation, and quality of life among the coastal communities in Sabah: Protocol of a population-based survey
Source: Medicine (Baltimore). 2020 Sep 11;99(37):e22067. doi: 10.1097/MD.0000000000022067 (PMC7489655; doi:10.1097/MD.0000000000022067)
Supplement: Supplemental Digital Content [file medi-99-e22067-s002.pdf]

BLUE COMMUNITIES SURVEY (HEAD OF HOUSEHOLD)

Coordinate	District	Village	House	Occupant	Interviewer

Part A: HOUSING AND THE ENVIRONMENT (Head of Household Only)

No	Description	Criteria/ Method	Options*

a) FAMILY STRUCTURE

1	**Jumlah ahli keluarga yang tinggal di dalam rumah ini.**<br>*Number of family members living in the house.*	Open-ended	1 – 99

b) HOUSING

2	**Apakah jenis rumah?**<br>*What type of house?*	Observation	0= Bungalow/detached house<br>1= Semi-detached<br>2= Row/Terrace house<br>3= Others including flats/condos
3	**Rumah itu di bina daripada ….**<br>*The house is constructed out of ….*	Observation	0=Brick/concrete<br>1=Wood<br>2=Wood and Bricks/concrete<br>3=Others
4	**Nyatakan jenis industri yang berhampiran dengan rumah ini**<br>*State the type of industry nearest to the house*	Observation	0=Cottage<br>1=Factory<br>2=Farms<br>3=Plantation
5	**Adakah kereta/ambulan dapat sampai ke rumah ini?**<br>*Is the house accessible by car / ambulance?*	Observation	0= No<br>1= Yes
6	**Apakah punca elektrik?**<br>*What is the source of electricity?*	Observation	0=Others<br>1=SESB
7	**Tanah ini di miliki oleh siapa?**<br>*Who is the owner of the land?*	Head of Household	1=Self/family<br>2=Rented/leased from individual/company<br>3=Government<br>4=Others<br>7=NA,<br>8=DK
8	**Rumah ini di miliki oleh siapa?**<br>*Who is the owner of this house?*		
9	**Berapakah bilangan bilik selain daripada bilik mandi/tandas?**<br>*How many rooms are there in the house **excluding** bathroom/toilets?*		1 - 99

NA= Not Applicable/available; DK=Don't Know

Di dalam rumah ini, adakah terdapat ....
In this house is there …

10	**TV /** *Television*	Head of Household / Observation	0=No<br>1=Yes
11	***Radio/*** *Radio*		
12	**Telefon (termasuk telefon bimbit)/** *Telephone (including mobile phone)*		
13	**Komputer (termasuk komputer riba)/** *Computers (including laptop)*		
14	***Kemudahan internet (termasuk data bimbit)/*** *Internet access (including mobile data)*		
15	***ASTRO/*** *ASTRO*		

c) HOUSEHOLD TRANSPORTATION

	(Family transportation means all vehicles owned by any members staying in the same house)		
16	**Adakah keluarga ini memiliki kereta?**<br>*Does the family own a car?*	Head of Household	0=No<br>1=Yes
17	**Adakah keluarga ini memiliki motor?**<br>*Does the family own a motorbike?*		
18	**Adakah keluarga ini memiliki lain-lain kenderaan?**<br>*Does the family own other vehicle?*		……………………..<br>(Other types of vehicle)

d) EXCRETA DISPOSAL

Apakah jenis tandas yang terdapat di rumah ini dan bagaimanakah keadaannya?
What are the type of toilets available in this house and the condition?

19	a) **Tarik /** *Cistern-flush*		0=No<br>1=Yes
	b) **Siram /** *Pour-flush*		
	c) **Lain-lain /** *Others*		…………………..<br>(State the type)
20	**Tarik**<br>*Cistern-flush*	Observation	0=Clean,<br>1=Dirty,<br>7=NA<br>*(for each type listed, if it is available state clean or dirty, otherwise state NA)*
21	**Siram**<br>*Pour-flush*	Observation	
22	**Lain-lain**<br>*Others*	Observation	
23	**Dimanakah sisa kumbahan itu dibuangkan?**<br>*Where does the sludge discharge?*	Observation	0=Communal septic tank<br>1=Individual septic tank<br>2=Man-made hole<br>3=Open/river<br>4=Others

NA= Not Applicable/available; DK=Don't Know

No	Description	Criteria/ Method	Options*

e) SOLID WASTE DISPOSAL

24	**Adakah isi rumah ini mempunyai pengumpulan sampah yang kerap?**<br>*Do you have regular garbage collection for your household?*	Head of Household	0 = No (Go to Q26)<br>1 = Yes (Go to Q25)
25	**Jika Ya, berapa kerap?**<br>*If Yes, how often?*		0 = Once a week<br>1 = Twice a week<br>2 = Three times a week<br>3 = Most days of the week<br>4 = Everyday
26	**Jika Tidak, bagaimanakah sisa sampah isi rumah ini dibuangkan?**<br>*If no, how do you dispose your household garbage?*		0 = Municipal Waste<br>1 = Open hole<br>2 = Burried<br>3 = Into the drain/river<br>4 = Along with the sludge<br>5 = Others (Burn etc.)
27	**Adakah anda asingkan jenis sisa-sisa buangan di rumah anda? (Kering/Basah, kertas, plastik, sisa makanan)**<br>*Do you separate different types of waste at your home? (For example: Dry/Wet, paper, plastics, food waste)*		0 = No<br>1 = Yes
28	**Bagaimanakah sisa dapur dibuangkan?**<br>*How is the kitchen waste disposed?*	Head of Household	0=Municipal waste<br>1=Open hole<br>2=Buried<br>3=Into the drain /river<br>4=Along with the sludge<br>5=Others (Burn etc.)
29	**Bagaimanakah sisa-sisa lain dibuangkan?**<br>*How is the other waste disposed?*	Head of Household	0=Municipal waste<br>1=Open hole<br>2=Buried<br>3=Into the drain /river<br>4=Along with the sludge<br>5=Others (Burn etc.)

f) WATER SUPPLY

	Drinking and cooking water		
30	**Apakah punca utama air yang digunakan untuk memasak dan minum?**<br>*What is the main source of drinking and cooking water?*	Head of Household	0=Pipe water<br>1=Shallow well water<br>2=Deep well water<br>3=Rain water<br>4=River water<br>5=Gravity-feed water (GFS)<br>6=Others

NA= Not Applicable/available; DK=Don't Know

31	**Bagaimanakan ianya dirawat?**<br>*How is it treated?*	Head of Household	1=Filtered<br>2=Reverse osmosis<br>3=Chlorination<br>4=Not treated<br>8=DK
32	**Adakah anda memasak air minuman anda?**<br>*Do you boil your drinking water?*	Head of Household	0=No<br>1=Yes
	Bathing and Washing		
33	**Apakah punca utama air yang digunakan untuk mandi dan mencuci?**<br>*What is the main source of water for washing and bathing?*	Head of Household	0=Pipe water<br>1=Shallow well water<br>2=Deep well water<br>3=Rain water<br>4=River water<br>5=Gravity-feed water (GFS)<br>6=Others

PART B: HOUSEHOLD INCOME

34	**Berapakah pendapatan anda sebulan dari pekerjaan bergaji?**<br>*How much did you earn a month from paid employment?*		0=not in paid employment<br>RM_____per month
35	**Berapakah pendapatan anda sebulan dari hasil kerja sendiri?**<br>*How much did you earn a month from self-employment?*		0=not in self-employment<br>RM_____per month
36	**Berapa banyak pencen yang anda terima sebulan?**<br>*How much pension do you receive a month?*		0=not receiving any pension<br>RM_____per month
37	**Selain daripada yang tersebut di atas, berapakah pendapatan anda dari sumber-sumber yang lain (seperti hasil sewa rumah/kedai/tanah, hasil jualan kebun, sumbangan daripada anak-anak/ibu/bapa atau lain-lain sumber)**<br><br>*In addition to those mentioned above, how much do you earn a month from other income sources (example from rental of properties, sales of produce from your farm, contribution from your children/father/mother or from other sources)?*		(Amount to the nearest RM)<br><br>RM______per month
38	**Berapakah jumlah pendapatan isi rumah***<br>*How much is the total household income?**		RM …………………..<br>*For head of household

NA= Not Applicable/available; DK=Don't Know

PART C: SOCIO-DEMOGRAPHIC (All Household Members)

No	Description	Criteria/ Method	Options*
39	**Tarikh lahir anda**	Open ended	....../....../.......
40	**Berapakah umur anda**	Open ended	....
41	**Apakah jantina anda?**<br>*What is your sex / gender?*	All	0=Male<br>1=Female
42	**Apakah etnik anda?**<br>*What is your ethnicity?*<br>*(based on the race of the father)*	All	0=Malay<br>1=Chinese<br>2=Indian<br>3=Other Bumiputra<br>4=Others
43	**Apakah agama yang anda menganuti?**<br>*Which religion do you practice?*	All	0=Islam,<br>1=Christian,<br>2=Buddhist,<br>3= Hindu,<br>4=Others,<br>5=Atheist<br>8=DK

a) SCHOOLING INFORMATION

44	**Pernahkah anda menghadiri sekolah?**<br>*Have you ever attended school?*	≥ 6 years	0=No,<br>1=Yes<br>7=NA
45	**Apakah tahap tertinggi persekolahan anda?**<br>*What is the highest level of education you have completed?*	≥ 6 years	0=Primary,<br>1=Secondary,<br>2=Certificate/Skill, (Post Secondary)<br>3=College/University<br>7=NA

b) MARRIAGE INFORMATION

46	**Apakah status perkahwinan anda?**<br>*What is your marital status?*	≥ 18 years	0=Single/Never married,<br>1=Married,<br>2=Divorce/separated,<br>3=Widowed
47	**Berapakah bilangan anak anda?**<br>*How many children do you have?*	≥ 18 years	(*Actual number of children)*<br>77=NA (not married)

NA= Not Applicable/available; DK=Don't Know

c) OCCUPATIONAL HISTORY

48	**Adakah anda berkerja sekarang atau dalam masa 6 bulan ini?**<br>*Are you currently working now or within the past 6 months?*	If No,<br>go to Q56	0=No,<br>1=Yes<br>7=NA (< 18 years)
49	*(For those who are currently working)*<br>**Apakah pekerjaan utama anda?**<br>*What is your main occupation?*	If Q41=1;<br>Open ended	……………………
50	**Siapakah majikan anda?**<br>*Who is your employer?*	If Q41=1	0=Government/Government linked<br>1=self employed / relatives<br>2=Local company<br>3=International company<br>7=NA (< 16 years; ≥ 16 years who have never worked)<br>8=DK
51	**Apakah sektor utama pekerjaan anda?**<br>*What is the main sector your occupation is involved?*	observation	0=Manufacturing<br>1=Agriculture<br>2=Fisheries<br>3=Others<br>77=NA (< 16 years; ≥ 16 years who have never worked)<br>88=DK9
52	**Dari bilakah anda mula berkerja dengan majikan tersebut?**<br>*When did you start working with your present employer?*	If Q41=1	Year: ……….
53	**Sekiranya anda sedang berkerja, berapa pekerjaan yang anda lakukan?**<br>*If you are currently working, how many jobs do you have?*		0=One,<br>1=Two,<br>2=Three<br>3=more than three,<br>7=NA (< 16; for those who are currently not working)
54	**Secara purata, berapakah jumlah jam anda bekerja dalam sehari?**<br>*On average, how many hours do you work in a day?*		____hours
55	**Secara purata berapakah hari anda berkerja dalam satu minggu?**<br>*On average, how many days do you work per week?*		____days/week

NA= Not Applicable/available; DK=Don't Know

PART D: EXPENDITURE AND UTILIZATION OF HEALTH CARE GOODS AND SERVICES

A) OUTPATIENT CARE

**56. Dalam 4 minggu yang lepas pernahkah anda mendapat rawatan pesakit luar dari hospital/ klinik / pengamal perubatan traditional / alternatif?**<br>*In the past 4 weeks, did you seek outpatient care in any hospital/clinic or from any traditional/alternative medical practitioner?*		0=No, go to Q67<br>1=Yes<br>8=DK

i) Sekiranya ya, berapa kalikah anda melawati tempat-tempat berikut:
If yes how many visits did you make to any of the following:

**57. Hospital kerajaan**<br>*Public hospital*		(Actual number of visits)<br>88=DK
**58. Hospital swasta**<br>*Private hospital*		
**59. Klinik kerajaan**<br>*Public clinic*		
**60. Klinik swasta**<br>*Private clinic*		
**61. Pengamal perubatan traditional / alternatif**<br>*Traditional/alternative medical practitioner*		

ii) Dalam masa 4 minggu yang lepas berapa jumlah telah dibayar untuk rawatan pesakit luar dari tempat-tempat berikut:
In the past 4 weeks how much did you ***pay for outpatient care*** *in any of the following:*

**62. Hospital kerajaan**<br>*Public hospital*		(Amount paid to the nearest RM)<br>8=DK
**63. Hospital swasta**<br>*Private hospital*		
**64. Klinik kerajaan**<br>*Public clinic*		
**65. Klinik swasta**<br>*Private clinic*		
**66. Pengamal perubatan traditional / alternatif**<br>*Traditional/alternative medical practitioner*		

NA= Not Applicable/available; DK=Don't Know

B) INPATIENT CARE

**67. Dalam 1 tahun yang lepas pernahkah anda dimasukkan ke hospital?**<br>*In the past 1 year, have you been admitted to any hospital?*		0=No, go to Q74<br>1=Yes<br>8=DK

Sekiranya ya, berapa kali anda pernah dimasukkan ke hospital-hospital berikut:
If yes, how many times have you been admitted to the following hospitals:

**68. Hospital kerajaan**<br>*Public hospital*		(Actual number of admissions)<br>8=DK
**69. Hospital swasta**<br>*Private hospital*		

Sekiranya ya, berapa hari anda dimasukkan ke hospital-hospital berikut:
If yes, how many days were you admitted to the following hospitals:

**70. Hospital kerajaan**<br>*Public hospital*		(Actual number of days admitted)<br>8=DK
**71. Hospital swasta**<br>*Private hospital*		

Dalam 1 tahun yang lepas berapa jumlah telah dibayar untuk rawatan pesakit dalam di hospital-hospital berikut?
In the past 1 year how much did you pay for inpatient care in any of the following hospitals?

**72. Hospital kerajaan**<br>*Public hospital*		(Actual amount in RM)<br>8=DK
**73. Hospital swasta**<br>*Private hospital*		

C) SELF-MEDICATION

**74. Dalam 4 minggu yang lepas, pernahkah anda membeli ubat sendiri tanpa berjumpa dengan mana-mana pengamal perubatan?**<br>*In the past 4 weeks, did you buy medicine without seeing any medical practitioner (**self-medication**)?*		0=No, go to Q76<br>1=Yes<br>8=DK
**75. Sekiranya ya, berapakah jumlah yang anda bayar sendiri untuk ubat-ubat itu?**<br>*If yes, how much did you actually pay for any self-medication using your own money?*		RM ………………….<br>(Amount paid to the nearest RM)<br>8=DK

NA= Not Applicable/available; DK=Don't Know

No	Description	Criteria/ Method	Options*

D) SOURCE OF HEALTH PAYMENTS

Siapakah yang bayar untuk rawatan kesihatan anda dalam setahun yang lepas?
Who paid for your health care during the past one year? (Answer all choices (multiple responses))

No	Description	Criteria/ Method	Options*
76	**Tiada bayaran dikenakan untuk rawatan**<br>*Free health care services*<br>*(Exm: Government officer, elderly (>65years old))*	If no, go to 83	0=No, 1=Yes<br>7=NA, 8=DK
77	**Sendiri/keluarga**<br>*Self/family*		0=No, 1=Yes<br>7=NA, 8=DK
78	**Majikan**<br>*Employer*		0=No, 1=Yes<br>7=NA, 8=DK
79	**Private Insurans**<br>*Insurance*		0=No, 1=Yes<br>7=NA, 8=DK
80	**Derma**<br>*Charity*		0=No, 1=Yes<br>7=NA, 8=DK
81	**Lain-lain (i.e – social insurances (PEKA B40)**<br>*Others*		0=No, 1=Yes<br>7=NA, 8=DK
82	**Berapa jumlah bayaran yang dikenakan dalam setahun yang lepas?**<br>*How much you/family paid for your health care treatment during the past one year?*		RM ....<br>(Open ended)

E) MISCELLANEOUS MEDICAL EXPENDITURES

No	Description	Criteria/ Method	Options*
83	**Dalam 1 tahun yang lepas, berapa jumlah yang telah dibelanjakan untuk mendapatkan rawatan lain? (Termasuk perubatan tradisi seperti bomoh)**<br><br>*In the past 1 year how much did you spend for* ***other healthcare****? (Including traditional healer such as sharman)*		(Actual amount in RM)<br>8=DK

NA= Not Applicable/available; DK=Don’t Know

PART E: MODIFIABLE LIFESTYLE FACTORS

a) TOBACCO CONSUMPTION

84	**Pernahkah anda menghisap tembakau (rokok, curut, shisha atau lain-lain)? (walaupun sekali)**<br>*Have you ever consumed tobacco products (cigarette, cigars, shisha etc)? (Even once)*	*If no. 5, go to Q95*	0=No, go to Q97<br>1= a few puffs *but* never smoked again<br>2= rarely<br>3=occasionally<br>4=Daily<br>5=Used to smoke regularly but have quit now
85	**Berapakah umur anda semasa anda mula merokok?**<br>*How old were you when you started smoking?*	*If Q84 ≠0, 1*	Age …….
86	**Kenapa anda merokok?**<br>*Why did you start smoking or why do you smoke?*	*If Q84 ≠0, 1*	0=peer pressure<br>1=curiosity/seek thrills/for fun<br>2=stress<br>3=weight control<br>4=stylish/cool/macho/ for appearance<br>5=role models (father/teacher/other adults)<br>6= mass media influence<br>7= Easily available/access<br>8=don't know<br>99=NA
87	**Berapa batang rokok yang anda hisap dalam seminggu ?**<br>*How many cigarettes do you smoke per week?*	*If Q84 ≠0, 1*	1-99
88	***(Only for current smokers)***<br>**Berapa yang anda belanjakan untuk rokok dalam seminggu?**<br>*How much do you spend on cigarettes in one week?*	*If Q84=2,3,4*	(Actual amount in RM)<br>888=DK<br>99
89	**Adakah anda berhajat untuk berhenti merokok?**<br>Did you attempt to quit smoking?		0=No<br>1=Yes
90	**Sekiranya ya, berapa banyak kalikah anda telah mencuba untuk berhenti merokok?**<br>*How many times did you attempt to quit smoking?*	*If Q89 = yes*	State the actual number of times<br>8=NA
91	**Sekiranya gagal untuk berhenti merokok, apakah sebab utama?**<br>*If failed attempt to quit, what was the main reason?*		1=withdrawal symptoms/nicotine dependency<br>2= weight gain<br>3=boring<br>4=stress<br>5=peer pressure<br>6=others (explain what)
92	**Adakah anda merasa bahawa tabiat anda merokok akan menjejaskan kesihatan orang yang tidak merokok?**<br>*Do you feel that your smoking habit jeopadise the health of non-smokers?*		0=No<br>1=Yes
93	**Dimanakah selalunya anda merokok?**<br>*Where do you smoke most?*		Open ended
94	**Dimanakah anda membuang puntung rokok?**<br>*Where do you throw the cigarette butt?*		
95	**Bilakah anda berhenti merokok?**<br>*How many months ago did you quit smoking?*	*If Q84 = 5*	In months……
96	**Apakah sebab utama anda berhenti merokok?**<br>*What was the main reason you quit smoking?*	*If Q84 = 5*	0=sebab-sebab kesihatan / health reasons<br>1=keluarga / family<br>2=kewangan / financial reason<br>3=agama / religion 7=NA 8=DK

NA= Not Applicable/available; DK=Don't Know

Environmental Tobacco Smoke (ETS)

ETS at HOME

97	**Dalam tempoh sehari, adakah anda terhidu asap rokok di rumah?**<br>*Were you exposed to cigarette smoke in a day at home?*		0=No,<br>1=Yes

ETS at WORK

98	**Dalam tempoh sehari, adakah anda terhidu asap rokok di tempat kerja?**<br>*Were you exposed to cigarette smoke in a day at work?*		0=No,<br>1=Yes<br>7=NA (for those not working)

ETS in SCHOOL

99	**Dalam tempoh sehari, adakah anda terhidu asap rokok di sekolah?**<br><br>*Were you exposed to cigarette smoke in a day in school?*		0=No,<br>1=Yes<br>7=NA (for those not going to school and teachers – for the case of teachers ETS exposure would be filled in the ETS at WORK question)

b) ASSESSMENT OF FOOD INTAKE PRACTICES

100	**Berapa kali anda makan dalam sehari?**<br>*How many times do you eat in a day?*		2=3 kali atau lebih sehari<br>1=2 kali sehari<br>0=Satu kali sehari
101	**Berapa kerap anda makan sarapan pagi?**<br>*How often do you eat breakfast?*		2=Setiap hari<br>1=1 – 2 kali seminggu<br>0=Sekali-sekala
102	**Biasanya sarapan pagi saya mengandungi: -**<br>*My breakfast usually contains: -*<br><br>*2 = Healthy food option*<br>*1 = Unhealthy food option*		2=Nasi/ bijirin sarapan/ roti bakar, buah dan minuman<br>1=Makanan bergoreng seperti mihun goreng, nasi goreng, roti canai, nasi lemak, telur dan minuman<br>0=Minuman sahaja
103	**Berapa kerap anda makan daging merah seperti daging lembu, kambing atau khinzir?**<br>*How often do you eat red meat such as beef, mutton or pork in a week?*		2=Kurang dari 4 kali seminggu<br>1=4 – 6 kali seminggu<br>0=Lebih daripada 6 kali seminggu
104	**Berapa kerap anda makan buah-buahan dan sayur-sayuran dalam sehari?** *How often do you eat fruits and vegetables in a day?*		2=3 kali sehari<br>1=1-2 kali sehari<br>0=Sekali-sekala
105	**Berapa kerap anda makan makanan manis (cth. gula-gula) pencuci mulut (dessert) yang manis atau berkrim, aiskrim atau coklat?**<br>*How often do you eat sweet or creamy desserts, ice cream or chocolate in a week?*		2=0 – 1 kali seminggu<br>1=2 – 4 kali seminggu<br>0=Lebih daripada 4 kali seminggu
106	**Berapa kerap anda makan ikan dalam seminggu?**<br>*How often do you eat fish in a week?*	*Fish refers to fresh fish only*	2=Lebih daripada 2 kali<br>1=1 – 2 kali<br>0=Sekali-sekala
107	**Berapa kerap anda makan makanan bergoreng?**<br>*How often do you eat fried foods in a week?*		2=Kurang daripada 3 kali seminggu<br>1=3 – 4 seminggu<br>0=Lebih daripada 4 kali seminggu

*JUMLAH SKOR: 12-16 =Cemerlang, 8-11=Baik, 4-7=Sederhana, 0-3=Kurang Memuaskan

NA= Not Applicable/available; DK=Don't Know

108	**Adakah anda minum susu?**<br>Do you take milk?		0=Tidak, go to Q110<br>1=Susu pekat manis<br>2=susu segar/tepung<br>3= Kedua-duanya
109	**Jika ya, berapa kerap?**<br>If yes, how often?		1=setiap hari<br>2=3-4 kali seminggu<br>3=Kurang daripada 3 kali seminggu

c) PHYSICAL ACTIVITY

International Physical Activity Questionnaire

Kami berminat untuk mengetahui aktiviti fizikal yang dilakukan oleh masyarakat umum dalam kehidupan harian mereka. Soalan-soalan berikut akan menyoal anda tentang jumlah masa yang anda gunakan untuk berada dalam keadaan aktif secara fizikal dalam tempoh 7 hari yang lepas ini. Sila jawab soalan-soalan ini walaupun anda berpendapat bahawa anda bukanlah seorang yang aktif. Sila fikirkan tentang aktiviti-aktiviti yang anda lakukan di tempat kerja, di rumah dan kawasan halaman, untuk bergerak dari satu tempat ke tempat yang lain, dan pada waktu lapang untuk rekreasi, senaman atau bersukan.

We are interested in finding out about the kinds of physical activities that people do as part of their everyday lives. The questions will ask you about the time you spent being physically active in the ***last 7 days****. Please answer each question even if you do not consider yourself to be an active person. Please think about the activities you do at work, as part of your house and yard work, to get from place to place, and in your spare time for recreation, exercise or sport.*

Fikirkan tentang semua aktiviti fizikal berat yang anda telah lakukan dalam tempoh 7 hari yang lepas ini. Aktiviti fizikal berat adalah aktiviti yang menggunakan daya tenaga fizikal yang kuat dan membuat anda bernafas jauh lebih kuat daripada biasa. Fikirkan hanya tentang aktiviti-aktiviti fizikal yang anda telah lakukan selama sekurang-kurangnya 10 minit pada sesuatu masa.

Think about all the ***vigorous*** *activities that you did in the* ***last 7 days****.* ***Vigorous*** *physical activities refer to activities that take hard physical effort and make you breathe much harder than normal. Think only about those physical activities that you did for at least 10 minutes at a time.*

110	**Dalam tempoh 7 hari yang lepas ini, berapa harikah anda telah melakukan aktiviti fizikal berat, contohnya mengangkat barang berat, mencangkul, senaman aerobik atau berbasikal laju?**<br><br>*During the* ***last 7 days****, on how many days did you do* ***vigorous*** *physical activities like heavy lifting, digging, aerobics, or fast bicycling?*	*If 'No' skip to Q-108*	_____ hari seminggu<br>0=No vigorous physical activity
111	**Berapakah masa yang anda biasa gunakan untuk melakukan aktiviti fizikal berat pada salah satu daripada hari berkenaan?**<br><br>*How much time did you usually spend doing* ***vigorous*** *physical activities on one of those days?*		_____minit sehari<br>8=DK (tidak tahu/tidak pasti)

Fikirkan tentang semua aktiviti fizikal sederhana yang anda telah lakukan dalam tempoh 7 hari yang lepas ini. Aktiviti fizikal sederhana adalah aktiviti yang menggunakan daya tenaga fizikal yang sederhana dan membuatkan anda bernafas agak lebih kuat daripada biasa. Fikirkan hanya tentang aktiviti-aktiviti fizikal yang anda telah lakukan selama sekurang-kurangnya 10 minit pada sesuatu masa.

Think about all the ***moderate*** *activities that you did in the* ***last 7 days****.* ***Moderate*** *activities refer to activities that take moderate physical effort and make you breathe somewhat harder than normal. Think only about those physical activities that you did for at least 10 minutes at a time.*

NA= Not Applicable/available; DK=Don't Know

112	**Dalam tempoh 7 hari yang lepas ini, berapa harikah anda telah melakukan aktiviti fizikal sederhana, contohnya mengangkat muatan ringan, mengelap lantai, berbasikal pada kelajuan biasa, atau bermain badminton beregu? Ini tidak termasuk berjalan kaki.**<br><br>*During the **last 7 days**, on how many days did you do **moderate** physical activities like carrying light loads, bicycling at a regular pace, or doubles tennis? **Do not include walking.***	*If 'No' skip to Q-110*	_____ hari seminggu<br>0=No moderate physical activity
113	**Berapakah masa yang anda biasa gunakan untuk melakukan aktiviti fizikal sederhana pada salah satu daripada hari berkenaan?**<br><br>*How much time did you usually spend doing **moderate** physical activities on one of those days?*		____minit sehari<br>8=DK (tidak tahu/tidak pasti)

Fikirkan tentang masa yang anda telah gunakan untuk berjalan kaki dalam tempoh 7 hari yang lepas ini. Masa ini merangkumi berjalan kaki di tempat kerja dan di rumah, berjalan kaki dari satu tempat ke tempat yang lain, dan berjalan kaki semata-mata untuk rekreasi, bersukan, bersenam atau pada masa lapang.

*Think about the time you spent **walking** in the **last 7 days**. This includes at work and at home, walking to travel from place to place, and any other walking that you might do solely for recreation, sport, exercise, or leisure.*

114	**Dalam tempoh 7 hari yang lepas ini, berapa harikah anda telah berjalan kaki selama sekurang-kurangnya 10 minit pada sesuatu masa?**<br><br>*During the **last 7 days**, on how many days did you **walk** for at least **10 minutes** at a time?*		_____ hari seminggu<br>99
115	**Berapakah masa yang anda biasa gunakan untuk berjalan kaki pada salah satu daripada hari berkenaan?**<br><br>*How much time did you usually spend **walking** on one of those days?*		____minit sehari<br>8=DK (tidak tahu/tidak pasti)

Soalan terakhir ini adalah berkaitan masa yang anda telah gunakan untuk duduk pada hari-hari bekerja dalam tempoh 7 hari yang lepas ini. Masukkan masa yang di habiskan duduk di tempat kerja, di rumah, sewaktu belajar dan di masa lapang. Masa ini juga merangkumi waktu yang di habiskan duduk di meja, menziarahi kawan-kawan, membaca, atau duduk atau baring sambil menonton televisyen.

*The last question is about the time you spent **sitting** on weekdays during the **last 7 days**. Include time spent at work, at home, while doing course work and during leisure time. This may include time spent sitting at a desk, visiting friends, reading, or sitting or lying down to watch television.*

116	**Dalam tempoh 7 hari yang lepas ini, berapakah masa yang anda telah gunakan untuk duduk pada sesuatu hari bekerja?**<br><br>*During the **last 7 days**, how much time did you spend **sitting** on a **week day**?*		____minit sehari<br>8=DK (tidak tahu/tidak pasti)

NA= Not Applicable/available; DK=Don't Know

d) MEDICAL CONDITION / DISEASES

Adakah ada ahli keluarga terdekat anda yang mempunyai penyakit-penyakit berikut?
Do any of your immediate family members have any of the following diseases?

117	**Kencing Manis**<br>*Diabetes Mellitus*		0=No, 1=Yes<br>2=DK
118	**Asma/Lelah**<br>*Asthma*		0=No, 1=Yes<br>2=DK
119	**Darah Tinggi**<br>*Hypertension*		0=No, 1=Yes<br>2=DK
120	**Sakit Jantung**<br>*Heart disease*		0=No, 1=Yes<br>2=DK
121	**Angin amar**<br>*Stroke*		0=No, 1=Yes<br>2=DK
122	**Barah**<br>*Cancer*	If No, go to 124	0=No, 1=Yes<br>2=DK
123	**Jenis barah**<br>*Type of cancer*		....

Pernahkan anda diberitahu oleh doktor anda bahawa anda menghidap penyakit-penyakit yang berikut?
Have you been informed by your doctor that you are suffering for the following disease?

No	Description	Criteria/ Method	Options*	No	Description	Options*
124	**Kencing Manis**<br>*Diabetes Mellitus*		0=No – Go to Q-126<br>1=Yes<br>7=NA	125	If Yes, are you taking any medication?	0=No<br>1=Yes<br>7=NA
126	**Asma/Lelah**<br>*Asthma*		0=No – Go to Q-128<br>1=Yes<br>7=NA	127	If Yes, are you taking any medication?	0=No<br>1=Yes<br>7=NA
128	**Darah Tinggi**<br>*Hypertension*		0=No – Go to Q-130<br>1=Yes<br>7=NA	129	If Yes, are you taking any medication?	0=No<br>1=Yes<br>7=NA
130	**Sakit Jantung**<br>*Heart disease*		0=No – Go to Q-132<br>1=Yes<br>7=NA	131	If Yes, are you taking any medication?	0=No<br>1=Yes<br>7=NA
132	**Angin amar**<br>*Stroke*		0=No – Go to Q-134<br>1=Yes<br>7=NA	133	If Yes, are you taking any medication?	0=No<br>1=Yes<br>7=NA
134	**Kolesterol tinggi**<br>*Hypercholesterolemia*		0=No – Go to Q-136<br>1=Yes<br>7=NA	135	If Yes, are you taking any medication?	0=No<br>1=Yes<br>7=NA
136	**Barah**<br>*Cancer*		0 = No - Go to Q-139<br>1 = Yes<br>7 = NA	137	If Yes, are you taking any medication?	0=No;<br>1=Yes;<br>7=NA
138	**Jika ya, apakah jenis barah?**<br>If Yes, what type of cancer?		0=No<br>1=Yes<br>7=NA			

NA= Not Applicable/available; DK=Don’t Know

e) INJURY

No	Description	Criteria/ Method	Options*
139	**Sejak setahun yang lepas pernahkah anda terlibat dengan sebarang kecederaaan atau kemalangan?**<br>*In the past **one-year** were you ever injured or involved in an accident?*	All	0=No – *Go to Q148 (Quality of Life)*<br>1=Yes
140	**Berapakah kali anda terlibat dengan kecederaan atau kemalangan dalam satu tahun yang lepas?**<br>*How many times were you injured or involved in an accident over the past one year?*		Actual number of times ....

Soalan berikut adalah untuk kemalangan dan kecederaan yang paling teruk yang anda alami,
The following questions is for the worst accident or injury that you were involved in,

No	Description	Criteria/ Method	Options*
141	**Apakah jenis kecederaan atau kemalangan?**<br>*What is the type of injury or accident?*		0= Not injured<br>1= Road traffic accident<br>2= poisoning<br>3= Choking<br>4= Fall<br>5= Burns/Scalding<br>6= Drowning<br>7= Injured by others<br>8= Injured oneself
142	**Dimanakah kecederaan / kemalangan itu berlaku?**<br>*Where did the injury / accident occur?*		0=Not injured<br>1=Home / Playground – *Go to Q143*<br>2=Work / School<br>3=Commuting / Road Traffic Accident – *Go to Q144*

No	Description	Criteria/ Method	Options*

Kemalangan di Rumah / *Residential Injury / Accidents*

No	Description	Criteria/ Method	Options*
143	**Jika kecederaan / kemalangan itu berlaku di rumah, dimanakah ia berlaku?**<br>*For the last home accident, in which part of the **house** did that accident occur?*	*Answer if Q142=1*	0=No home accident<br>1=Bilik air / Bathroom<br>2=Dapur/Kitchen<br>3=Ruang tetamu/ Living room<br>4=Bilik tidur/ Bedroom<br>5=Di atas tangga/On the Stairs<br>6=Di luar rumah/Outside compound<br>7=Di taman kanak-kanak / at the playground

Kemalangan Kenderaan Bermotor / *Motor Vehicle Accident*

No	Description	Criteria/ Method	Options*
144	**Jika kecederaan / kemalangan itu berlaku di jalanraya, apakah peranan anda pada masa itu?**<br>*If it was a road accident, what was your role at the time?*	*Answer if Q142=3*	0=No RTA accident<br>1=Bus / Lorry Driver<br>2=Bus / Lorry passenger<br>3=Motorist Driver<br>4=Motorist Occupant<br>5=Motorcycle Ride<br>6=Motorcycle Pillion rider<br>7=Cyclist<br>8=Pedestrian

NA= Not Applicable/available; DK=Don't Know

145	**Pada masa bilakah kecederaan / kemalangan yang paling teruk itu berlaku?**<br>*During what time of the day did the worst injury / accident occur?*		0= Tiada kemalangan / No accident<br>1= Pagi / morning - 6 am - 12 noon<br>2= Tengah hari / afternoon12 noon - 4 pm<br>3= Evening / petang - 4 pm - 8 pm<br>4= Night / malam - 8 pm-6 am
146	**Apakah jenis rawatan yang diterima selepas kecederaan itu?**<br>*What type of treatment did you seek after the injury?*		0=No Accident<br>1= No/Self treatment<br>2=Out-patient treatment in Govt clinic / hospital<br>3=Out-patient treatment in Private clinic / hospital<br>4=Traditional healer /alternative medicine<br>5= In-patient in Government hospital<br>6= In-patient treatment in Private hospital
147	**Apakah kesan daripada mana-mana kecederaan / kemalangan itu?**<br>*What is the final / current outcome of the injury / accident?*		0=No accident<br>1=Recovered completely<br>2=Still in recovery period<br>3=Under rehabilitation<br>4=Impaired / impairment

PART F: SF12 (QUALITY OF LIFE)

No	Description	Criteria/ Method	Options*
148	**Secara umum, adakah anda akan mengatakan bahawa kesihatan anda adalah:**<br>*In general, would you said your health is:*		1=Paling Baik / Best<br>2= Sungguh Baik/Very Good<br>3= Baik/Good<br>4=Sederhana/Medium<br>5= Tidak Baik/Not Good
**Soalan-soalan berikut adalah mengenai aktiviti yang mungkin akan dilakukan oleh anda pada hari biasa. Adakah <u>kesihatan anda sekarang menghadkan anda</u> dalam aktiviti-aktiviti berikut? Jika ya, sejauh mana? Jika ya, sejauh mana?**<br>*The following items are about activities you might do during a typical day. Does <u>your health now limit</u> you in these activities? If so, how much?*			
149	**Aktiviti sederhana, seperti mengalihkan meja, menyapu, bermain badminton atau bercucuk tanam**<br>*Simple activities, like moving tables, sweeping, playing badminton or planting*		0=Ya, Terbatas dengan banyaknya/ Yes, major limitation<br>1= Ya, terbatas dengan sedikitnya/ Yes, limited limitation<br>2=Tidak terbatas sama sekali/No limitation at all
150	**Menaiki beberapa tingkat tangga?**<br>*Climbing several flights of stairs?*		0=Ya, Terbatas dengan banyaknya/ Yes, major limitation<br>1= Ya, terbatas dengan sedikitnya/ Yes, limited limitation<br>2=Tidak terbatas sama sekali/No limitation at all
**Dalam masa <u>4 minggu yang lalu</u>, berapa kerapkah anda mengalami sebarang masalah berikut dengan pekerjaan atau aktiviti harian tetap anda yang lain akibat daripada kesihatan fizikal anda?**<br>*During the <u>past 4 weeks</u>, have you had any of the following problems with your work or other regular daily activities <u>as a result of your physical health?</u>*			

NA= Not Applicable/available; DK=Don't Know

151	**Mencapai kurang daripada yang anda inginkan.**<br>*Accomplished less than what you would like.*		0= No<br>1= Yes
152	**Terbatas dari segi jenis pekerjaan atau aktiviti lain**<br>*Were limited in the kind of work or activities?*		0= No<br>1= Yes
**Dalam masa <u>4 minggu yang lalu</u>, pernahkah anda mengalami sebarang masalah berikut dengan pekerjaan atau aktiviti harian tetap anda yang lain <u>akibat daripada sebarang masalah emosi</u> (seperti merasa murung atau bimbang)?**<br>*During the <u>past 4 weeks</u>, have you had any of the following problems with your work or others regular daily activities <u>as a result of any emotional problems</u> (such as feeling depressed or anxious)*			
153	**Mencapai kurang daripada yang anda inginkan**<br>*Accomplised less than you would desire*		0= No<br>1= Yes
154	**Melakukan pekerjaan atau aktiviti lain dengan kurang berhati-hati daripada biasa**<br>*Doing other work or activities with less caution than usual*		0= No<br>1= Yes
155	**Dalam masa <u>4 minggu yang lalu,</u> sejauh manakah <u>kesakitan</u> telah mengganggu pekerjaan biasa anda (termasuk pekerjaan di luar rumah dan kerja rumah)?**<br>*Within the <u>past 4 weeks,</u> how much <u>pain</u> interfere with your normal work (including work outside your home and housework)?*		0= Tidak sama sekali/Not atall<br>1= Sedikit/A little bit<br>2= Sederhana/Moderately<br>3 = Lebih daripada biasa/Quite a bit<br>4= Sangat tinggi/Extremely
**Soalan-soalan ini adalah mengenai perasaan dan keadaan anda dalam masa <u>4 minggu yang lalu</u>. Untuk setiap soalan, sila berikan satu jawapan yang paling hampir dengan keadaan perasaan anda. Dalam masa <u>4 minggu yang lalu,</u> berapa kerapkah: -**<br><br>*These questions are about how you feel and how things have been with you <u>during the past 4 weeks.</u> For each question, please give the one answer that comes closest to the way you have been feeling. How mch of the time during the <u>past 4 weeks</u>:-*			
156	**Adakah anda merasa tenang dan aman?**<br>*Have you felt calm and safe?*		0= Setiap masa/ all the time<br>1= Kebanyakan masa/ most of the time<br>2=Kadang-kala/ Sometimes<br>3= Sedikit masa/ Seldom<br>4=Tidak sama sekali/ None at all
157	**Adakah anda sungguh bertenaga?**<br>*Didyou have a lot of energy?*		0= Setiap masa/ all the time<br>1= Kebanyakan masa/ most of the time<br>2=Kadang-kala/ Sometimes<br>3= Sedikit masa/ Seldom<br>4=Tidak sama sekali/ None at all
158	**Pernahkah anda merasa sedih dan murung?**<br>*Have you ever downhearted and blue?*		0= Setiap masa/ all the time<br>1= Kebanyakan masa/ most of the time<br>2=Kadang-kala/ Sometimes<br>3= Sedikit masa/ Seldom<br>4=Tidak sama sekali/ None at all
159	**Dalam masa <u>4 minggu yang lalu</u>, berapa kerapkah <u>kesihatan fizikal atau masalah emosi</u> telah mengganggu aktiviti sosial anda (seperti melawat sahabat-handai, sanak-saudara, dll.)?**<br>*During the <u>past 4 weeks,</u> how often has your physical health or emotional problems interfered with your social activities (such as visiting friends, relatives, etc.)?*		0= Setiap masa/ all the time<br>1= Kebanyakan masa/ most of the time<br>2=Kadang-kala/ Sometimes<br>3= Sedikit masa/ Seldom<br>4=Tidak sama sekali/ None at all

NA= Not Applicable/available; DK=Don't Know

PART G: INTERACTION WITH MARINE LOCAL ENVIRONMENT (160)

Pada bahagian ini, kami ingin anda memerlukan sedikit lagi maklumat tentang jenis-jenis aktiviti yang anda lakukan dalam dan sekitar persisiran pantai dalam satu minggu yang lepas:
In this section we would like you to say a little bit more on the kinds of activities you have done in and around the coast in the last week:

No	Description	Criteria/ Method	Options*
**Penghidupan** / *Livelihood*		Number of days	
a	**Pergi menangkap ikan untuk dimakan atau sumber kewangan**<br>*Went wild fish fishing for food or to earn money*		
b	**Pergi menangkap ketam/udang dll.**<br>*Went shellfish fishing for crab/shrimp etc.*		
c	**Pergi memungut cengkerang ketika air surut**<br>*Went gleaning (e.g. shells) at low tide*		
d	**Menjalankan aktiviti akuakultur**<br>*Performed aquaculture activities*		
e	**Menjalankan aktiviti pertanian rumpai laut**<br>*Performed seaweed farming activities*		
f	**Menjalankan aktiviti selepas penangkapan (Cth: pengeringan ikan)**<br>*Performed post-harvest activities (e.g. fish drying)*		
g	**Mengumpul nipah sawit**<br>*Nypa palm collection*		
h	**Mengumpul makanan liar/asli (Cth: madu)**<br>*Wild food collection (e.g. honey)*		
i	**Menjual hasil**<br>*Sold produce*		
j	**Membawa pelancong menaiki bot**<br>*Taken tourists on a boat*		
**Kehidupan Harian/***Day-to-day living*		Number of days	
k	**Membasuh diri/anak-anak di laut**<br>*Washed self/children in the sea*		
l	**Membasuh pakaian/pinggan mangkuk di laut**<br>*Washed clothes/dishes in the sea*		
m	**Makan ikan segar daripada laut**<br>*Eaten fresh fish from the bay*		
n	**Makan kerang-kerangan segar daripada laut**<br>*Eaten fresh shellfish from the bay*		
o	**Menebang paya/Membuat arang**<br>*Cut mangroves/made charcoal*		
p	**Pernah menaiki bot sebagai pengangkutan (untuk pergi ke pulau)**<br>*Been on a boat for transport (to get to an island)*		
q	**Pergi berenang/snorkeling untuk berseronok**<br>*Went swimming/snorkelling for fun*		
r	**Terlihat mamalia/burung/reptilia marin**<br>*Saw marine mammals/birds/reptiles*		
s	**Pergi ke persisiran pantai untuk bermain/rekreasi**<br>*Went along the shoreline for play/recreation*		
		Number of days	

NA= Not Applicable/available; DK=Don't Know

Pengurusan persekitaran / *Environmental management*			
t	**Mengadakan pembersihan/memungut sampah di pantai**<br>*Did a beach clean/litter pick*		
u	**Pemuliharaan marin (Cth: Menanam bakau baru)**<br>*Marine conservation (e.g. planted new mangroves)*		
v	**Menjalankan rondaan berkaitan marin**<br>*Conducted marine related patrols*		
w	**Mengambil bahagian dalam perbincangan/inisiatif pihak berkepentingan**<br>*Took part in stakeholder discussions/initiatives*		

PART H: PERCEPTIONS OF THE MARINE ENVIRONMENT IN TMP

Pada bahagian ini, kami ingin anda memikirkan tentang TMP, bagaima ianya telah berubah dalam 10 tahun yang lepas, dan apa yang anda fikir akan terjadi dalm 10 tahun mendatang. Secara tepatnya, kami ingin anda fikirkan tentang aktiviti-aktiviti berikut:
In this section we would like you to think about TMP, how has it changed over the last ten years, what do you think will happen in the next 10 years. In particular we would like you to think of the following activities:

No	Description	Criteria/ Method (Scale 1-7) 1 = Good/More → 7=Worst/Less	161 10 tahun lepas/10 years ago	162 10 tahun akan datang /10 years from now
**Sumber /** Resources				
a	**Jumlah (nombor) ikan liar, kepelbagaian jenis ikan**<br>*Amount (number) of wild fish, diversity of fish types*	**(Scale 1-7)**		
b	**Jumlah hidupan bercengkerang liar, kepelbagaian jenis hidupan bercengkerang**<br>*Amount of wild shellfish, diversity of shellfish types*	**(Scale 1-7)**		
c	**Jumlah ikan akuakultur (Cth: Ikan dalam sangkar)**<br>*Amount of fish aquaculture (e.g. fish cages)*	**(Scale 1-7)**		
d	**Jumlah hidupan bercengkerang dalam akuakultur (e.g. kepah)**<br>*Amount of shellfish aquaculture (e.g. mussel lines)*	**(Scale 1-7)**		
e	**Jumlah pertanian rumpai laut**<br>*Amount of seaweed farming*	**(Scale 1-7)**		
**Habitat**/Habitats				
f	**Kualiti terumbu karang, kepelbagaian jenis terumbu karang**<br>Quality of coral reefs, diversity of coral types	**(Scale 1-7)**		
g	**Liputan rumpai laut, jenis spesis rumpai laut**<br>Seagrass coverage, number of seagrass species	**(Scale 1-7)**		
h	**Liputan paya bakau, kepelbagaian jenis paya bakau**<br>Mangrove coverage, diversity of mangrove types	**(Scale 1-7)**		
i	**Liputan pohonan pantai yang lain**<br>Other beach tree cover	**(Scale 1-7)**		
**Kualiti air/**Water quality				
j	**Jumlah racun perosak pertanian di dalam air**<br>*Amount of farming pesticides/fertilisers in the water*	**(Scale 1-7)**		
k	**Jumlah/Kepekatan sisa kumbahan di dalam air**	**(Scale 1-7)**		

NA= Not Applicable/available; DK=Don't Know

	Amount/Concentration of sewage in the water			
l	**Jumlah plastic/sampah di dalam air**<br>*The amount of plastics/rubbish n the water*	**(Scale 1-7)**		
m	**Warna dan bau air laut**<br>*The colour and smell of the sea water*	**(Scale 1-7)**		
n	**Rasa ikan/cengkerangan daripada air ini**<br>*The taste of fish/shellfish from these waters*	**(Scale 1-7)**		
o	**Frekuensi ledakan alga berbahaya**<br>*The frequency of Harmful Algal Blooms*	**(Scale 1-7)**		
p	**Bekalan air minuman yang bersih**<br>*The supply of clean drinking water*	**(Scale 1-7)**		
**Isu-isu lain**/Other issues				
q	**Isu lain yang anda ingin bincangkan**<br>*Other issues you would like to discuss*	Open ended		

NA= Not Applicable/available; DK=Don't Know

PART I: MEDICAL PROFORMA

No	Description	Criteria/ Method	Options*
163	**Ukuran berat sebenar (kg)** / *Weight (kg)*	*Use the medical equipments*	Actual weight
164	**Ukuran tinggi sebenar (cm)** / *Height (cm)*		Actual height
165	**Ukur lilit pinggang (cm)** / *Waist circumference (cm)*		Actual measurement
166	**Ukur lilit pinggul (cm)** / *Hip circumference (cm)*		Actual measurement

Bacaan Tekanan Darah/*Blood Pressure Measurement*

	**Bacaan pertama**/1st Reading		
167	**Bacaan tekanan darah sistolik**/Systolic reading (SBP1)		Actual mesurement
168	**Bacaan tekanan darah diastolik**/Diastolic reading (DBP1)		Actual mesurement
	***Bacaan kedua**/2nd Reading		
169	**Bacaan tekanan darah sistolik**/Systolic reading (SBP2)		Actual mesurement
170	**Bacaan tekanan darah diastolik**/Diastolic reading (DBP2)		Actual mesurement
	****Bacaan ketiga**/3rd reading		
171	**Bacaan tekanan darah sistolik**/Systolic reading (SBP3)		Actual mesurement
172	**Bacaan tekanan darah diastolik**/Diastolic reading (DBP3)		Actual mesurement

***There must be AT LEAST 1 (ONE) minute gap between 1st and 2nd reading of the blood pressure**
**** Third reading only needs to be performed when the blood pressure was high (systolic BP > 140) in either first or second reading or even both readings.**
Standard Operation Procedure for Blood Pressure Collection

1. Take two readings. If the first reading for systolic is less than 140, wait for 1 minute, then take the second reading. If the second reading for systolic is also less than 140, then record both readings in the answer booklet.
2. Take two readings. If either of the two readings of systolic is high or both are high, then wait for 30 minutes. Then, take the third reading. If the third reading of systolic is high then write a referral letter to the nearest hospital. If the third reading of systolic is less than 140, then no need to write a referral letter. However, the interviewer must advice the participants to check at the clinic/hospital in the future. All the readings must be recorded in the answer booklet.

NA= Not Applicable/available; DK=Don't Know
