# Supplementary material for: Health status, healthcare utilisation, and quality of life among the coastal communities in Sabah: Protocol of a population-based survey
Source: Medicine (Baltimore). 2020 Sep 11;99(37):e22067. doi: 10.1097/MD.0000000000022067 (PMC7489655; doi:10.1097/MD.0000000000022067)
Supplement: Supplemental Digital Content [file medi-99-e22067-s003.doc]

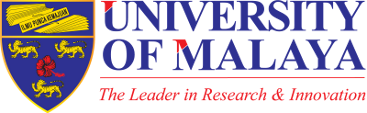


**Lampiran 1:**

**KERTAS INFORMASI PESERTA**

**Tajuk Kajian:** “Projek 6: Faedah Kesejahteraan dan Risiko Kehidupan Persisiran Pantai”

**Pengenalan:**

Kepada peserta yang dihormati,

Adalah penting untuk mengetahui tentang tahap kesihatan penduduk, keperluan dan penggunaan perkhidmatan kesihatan bagi sesuatu kawasan supaya intervensi yang sesuai dapat dikenal pasti dan diimplementasikan untuk kesejahteraan penduduk. Sehubungan itu, satu kajian soal selidik akan dilakukan di kawasan tuan/puan daripada November 2019 hingga April 2020. Tuan/puan akan dilawati oleh dua orang penyelidik dan dijemput untuk menyertai kajian ini dengan menjawab soal selidik yang akan diadakan. Selain daripada itu, pemeriksaan kesihatan seperti mengambil ukuran berat badan dan tekanan darah serta pemerhatian ke atas persekitaran tempat tinggal juga akan dilakukan oleh penyelidik. Semua data-data yang diperolehi daripada kajian ini adalah dianggap sebagai sulit dimana data-data hanya akan digunakan tanpa merujuk kepada sebarang individu.

**Penyertaan dalam kajian ini:**

• Penyertaan anda dalam kajian ini adalah secara sukarela.

• Anda boleh enggan mengambil bahagian dalam kajian ini atau anda boleh menarik diri daripada penyertaan anda pada bila-bila masa tanpa penalti.

• Sekiranya terdapat sebarang soalan yang anda dapati terlalu sensitif, anda boleh tidak menjawabnya atau meminta untuk menghentikan perbincangan.

• Jawapan dan maklumat anda akan dirahsiakan oleh penyelidik kajian dan tidak akan diumumkan kepada umum kecuali undang-undang Malaysia memerintahkannya.

• Dengan menandatangani borang persetujuan, anda akan memberi kami kebenaran untuk menyemak rekod, menganalisis dan menggunakan data yang dikumpulkan dalam kajian ini.

**Faedah Kajian ini:**

- Data yang dikumpul daripada kajian ini dapat memberi gambaran tentang tahap kesihatan penduduk di kawasan persisiran pantai Taman Tun Mustapha.
- Hasil daripada analisis data boleh digunakan untuk menentukan intervensi yang diperlukan oleh penduduk di kawasan ini.
- Peserta yang dikenalpasti mempunyai masalah kesihatan akan dirujuk untuk pemeriksaan lanjut di fasiliti kesihatan yang berdekatan.

**Risiko Kajian:**

Tiada risiko kajian yang membimbangkan kerana tiada pengambilan darah yang akan dilakukan dan data-data yang dikumpul, dianalisa dan dibentangkan tidak akan merujuk kepada seseorang individu.

**Maklumat lanjut dan butiran perhubungan:**

Sekiranya tuan/puan bersetuju untuk menyertai kajian ini, sila tandatangani borang persetujuan untuk menyertai kajian. Sebarang pertanyaan bolehlah menghubungi penyelidik-penyelidik yang berikut;

1. Prof. Dr. Maznah Binti Dahlui

Universiti Malaya, Kuala Lumpur

Email: maznahd@ummc.edu.my

No. Tel: 03-79492108

1. Dr. Mohd Aizat Mohd Zain

Universiti Malaya, Kuala Lumpur

Email: aizat03@gmail.com

No. Tel: 03-79677513

1. Dr. Mohd Iqbal Mohd Noor

Universiti Malaya, Kuala Lumpur

Email: mohdiqbalmohdnoor@gmail.com
